# Seeing the forest through the trees: prioritising potentially functional interactions from Hi-C

**DOI:** 10.1186/s13072-021-00417-4

**Published:** 2021-08-28

**Authors:** Ning Liu, Wai Yee Low, Hamid Alinejad-Rokny, Stephen Pederson, Timothy Sadlon, Simon Barry, James Breen

**Affiliations:** 1 Computational & Systems Biology, Precision Medicine Theme, South Australian Health & Medical Research Institute, SA 5000 Adelaide, Australia; 2 Robinson Research Institute, University of Adelaide, SA 5005 Adelaide, Australia; 3 Adelaide Medical School, University of Adelaide, SA 5005 Adelaide, Australia; 4 The Davies Research Centre, School of Animal and Veterinary Sciences, University of Adelaide, Roseworthy, SA 5371 Australia; 5 BioMedical Machine Learning Lab, The Graduate School of Biomedical Engineering, The University of New South Wales, NSW 2052 Sydney, Australia; 6 Core Member of UNSW Data Science Hub, The University of New South Wales, 2052 Sydney, Australia; 7 Women’s & Children’s Health Network, SA 5006 North Adelaide, Australia; 8 South Australian Genomics Centre (SAGC), South Australian Health & Medical Research Institute (SAHMRI), SA 5000 Adelaide, Australia; 9 Dame Roma Mitchell Cancer Research Laboratories (DRMCRL), Adelaide Medical School, University of Adelaide, SA 5005 Adelaide, Australia

**Keywords:** Chromosome conformation capture, Hi-C, Statistically significant interactions identification, Data integration

## Abstract

Eukaryotic genomes are highly organised within the nucleus of a cell, allowing widely dispersed regulatory elements such as enhancers to interact with gene promoters through physical contacts in three-dimensional space. Recent chromosome conformation capture methodologies such as Hi-C have enabled the analysis of interacting regions of the genome providing a valuable insight into the three-dimensional organisation of the chromatin in the nucleus, including chromosome compartmentalisation and gene expression. Complicating the analysis of Hi-C data, however, is the massive amount of identified interactions, many of which do not directly drive gene function, thus hindering the identification of potentially biologically functional 3D interactions. In this review, we collate and examine the downstream analysis of Hi-C data with particular focus on methods that prioritise potentially functional interactions. We classify three groups of approaches: structural-based discovery methods, e.g. A/B compartments and topologically associated domains, detection of statistically significant chromatin interactions, and the use of epigenomic data integration to narrow down useful interaction information. Careful use of these three approaches is crucial to successfully identifying potentially functional interactions within the genome.

## Background

The three-dimensional (3D) architecture of the eukaryotic genome has been shown to be an important factor in regulating transcription [1–3]. In the nucleus, DNA is folded into a highly organised structure, allowing transcriptional and regulatory machinery to be in specific nuclear territories for efficient usage. The impact of DNA folding and the resulting physical interactions can have dramatic impacts on the regulation of the genes, enabling non-coding regions such as regulatory elements (e.g. enhancers and silencers) to act on distally located gene promoters with disruption of chromosomal organisation increasingly linked to disease [4–6]. However, while highly organised, the folding structure of the 3D genome can also be highly dynamic to allow for the flexibility and modularity to facilitate regulatory action across a wide-range of cell types and biological processes, such as development, immune homeostasis, cancer and diseases.

In recent decades, the development of chromosome conformation capture assays and high-throughput sequencing has facilitated the construction of 3D genomes at high resolution, enabling the identification of cell type and tissue-specific 3D interactions between regions in the genome. However, the analysis of such data is complicated by the massive amount of identified physical interactions, hindering the detection and interpretation of interactions that are biologically meaningful. In this review, we introduce the background of 3D genome structure and its components, followed by a summary of the protocols that are commonly used to study 3D genome architecture in recent years, focusing on Hi-C protocols and other derived methods, whilst the use of microscopy to image 3D genome organisation has also been recently reviewed [7]. We then thoroughly review current in silico methods for identification of potentially functional interactions, which are contacts with higher chance to be biologically functionally relevant, and categorise them into three methodological groups.

### Chromosome architecture and gene regulation

Within eukaryotic nuclei, chromosomal DNA is condensed and folded into highly organised 3D structures, with distinct functional domains [8, 9]. A key consequence of chromosome folding is that it can bring DNA regions that are far away from each other on the same linear DNA polymer (i.e. intra-chromosomal), into close proximity, allowing direct physical contact to be established between regions. Interchromosomal interactions may also play an important role in transcriptional regulation but are less studied. The best characterised examples of this type of interaction include the clustering of ribosomal genes to form the nucleolus and the clustering of olfactory receptor genes to ensure the monogenic and mono-allelic expression in an individual olfactory neuron [10].

**Fig. 1:**
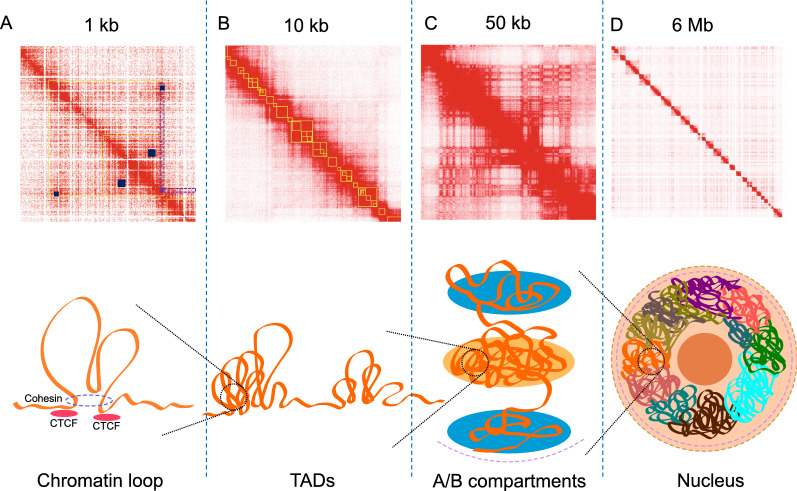
Illustration of genome architecture and the corresponding Hi-C interaction maps. Top panel: interaction heatmaps A, B, C, D are in different scales (kb or Mb per pixel) to correlate with the diagrams of 3D structures in the bottom panel, yellow boxes in A and B are identified TADs and small blue boxes in A indicate chromatin loops. The purple box in A is a frequently interacting region, with its classical “V” shape pattern coloured in purple dotted lines. Heatmaps were generated using Juicebox [29] with published Hi-C data of GM12878 [3]. Bottom panel: diagrams of 3D structures in the genome

The most basic level of chromosome organisation is chromatin “Loop” structures (Fig. 1A). Chromatin loops are formed based on a loop extrusion model, where linear DNA is squeezed out through the structural maintenance of chromosomes (SMC) cohesin complex until the complex encounters convergent CTCF bound at loop anchor sequences [8, 11–14]. Chromatin loops can either bring distal enhancers and gene promoters into close proximity to increase gene expression, or exclude an enhancer away from the loop to initiate boundaries to repress gene expression [15–17]. The archetypal chromatin looping factors are the CCCTC-binding protein (CTCF) and Cohesin complex [18–20], with the initial transient chromatin loops are created by the Cohesin complex during the extrusion process, or anchored on one CTCF binding site while the other anchor moving dynamically [11, 21, 22]. Moreover, specific transcription factors such as EKLF, GATA-1, FOG-1, NANOG and YY1 [23–28] were confirmed to play important roles in the regulation of chromatin looping.

Chromatin folding and DNA looping in particular leads to the formation of large-scale chromatin structures such as topologically associated domains (TADs) and chromosome compartments (Fig. 1B) [30]. TADs are defined by chromatin interactions occurring more frequently within the TAD boundaries, with TAD boundaries often demarcating a change in interaction frequency [30]. TAD boundaries are also enriched for the insulator-binding protein CTCF and cohesin complex [19, 20]. CTCF motif orientation appears to play a role in demarking TAD boundaries with some studies indicating that the majority of identified TADs (~ 60–90%) have a CTCF motif at both anchor boundaries with convergent orientation [3, 31, 32]. This is consistent with the loop extrusion model mentioned above, suggesting that the formation of most TADs are form by extrusion and are strictly confined by boundaries established by ‘architectural’ proteins such as CTCF and SMC cohesin complex [33], along with the boundaries engaging with strong 3D interactions [34]. Moreover, experimental inversion of CTCF orientation or complete removal of the CTCF binding sites have been shown to disrupt the formation or shift the boundary of a TAD [14, 16, 32], further emphasising the important role of CTCF defining TAD boundaries. The size of TADs are highly dependent on the resolution of the data and the chosen TAD caller and parameters [35], it can vary from hundreds of kilobases (kb) to 5 megabases (Mb) in mammalian genomes [36, 37], and also show significant conservation in related species [38], suggesting that they may serve as the functional base of genome structure and development. With higher sequencing depth, patterns of interactions across regions within a TAD can be further divided into “sub-TADs” with a median size of 185 kb using one kilobase resolution data [3], enabling finer scale investigation of the genome structure [39, 40]. In addition to “sub-TADs”, many other terms of TADs with different sizes and features have been proposed, including “micro-TADs” [41], “mega-domains” [42] and “super-TADs” [43]. However, functional distinction between the “conventional TADs” and them is still unclear. Evidence has shown that TADs are crucial structural units of long-range gene regulation [44–47], with interactions such as promoter–enhancer looping mostly found within the same TADs [48], and abnormal interactions across TADs (inter-TADs) can lead to significant regulation of expression level of important genes [49].

At a multi-megabase scale, the genome organisation is spatially segregated into euchromatin (gene-rich regions) or heterochromatin (gene-poor regions) to form active and inactive domains called ‘Compartments’ (Fig. 1C) [2]. This compartmentalisation of chromosome folding depicts the global organisation of chromosomes in the nucleus, where compartment A corresponds to gene-dense, euchromatic regions, and compartment B corresponding to gene-poor heterochromatin. Using higher resolution data, the genome can be further grouped into six sub-compartments, compartment A is separated into A1 and A2 whereas compartment B is separated into B1, B2, B3 and B4, with each one associated with specific histone marks [3]. Sub-compartments A1 and A2 are enriched with active genes and the activating histone marks H3K4me3, H3K36me3, H3K27ac and H3K4me1. Sub-compartments A1 and A2 are also depleted in nuclear lamina and nucleolus-associated domains (NADs). B1 domains correlate with H3K27me3 positively and H3K36me3 negatively, B2 and B3 are enriched in nuclear lamina but B3 is depleted in NADs, and B4 is an 11-Mb region, containing lots of KRAB-ZNF genes [3].

The interaction of transcription factors bound at regulatory elements, such as promoters, enhancers and super-enhancers, mediate the transcription level of a gene via interactions which are the direct result of the 3D chromosome structure, but which appear to be long-distance interactions when viewed through lens of a linear chromosome [50–52]. One early and well-characterised example is the interaction between beta-globin locus and its locus control region (LCR) [53]. During the development and differentiation of erythroid in human and mouse, the LCR, which is located 40–60 kb away from beta-globin genes, contains the hypersensitive sites that are exhibiting strong enhancer function and contacting to beta-globin genes distally via chromatin loops to regulation gene expressions [54–56]. *Hox* gene clusters, essential for patterning the vertebrate body axis, are also governed by a rich enhancer interaction network. Using chromatin conformation capture methods, a number of studies found that the transcriptional activation or inactivation of *Hox* clusters requires a bimodal transition between active and inactive chromatin [30, 57–60]. Taken together, the 3D genome structure governing long-distance contacts can build complex gene regulatory networks, allowing for either multiple enhancers to interact with a single promoter or a single enhancer to contact multiple promoters [61]. Disruption of these long-range regulatory networks is increasingly being linked to both monogenic and complex diseases [62, 63].

### Hi-C assays to quantify chromatin interactions

In order to investigate the 3D genome architecture, a series of protocols called chromosome conformation capture (3C) assays have been developed that specifically capture the physical interactions between regions of DNA [1, 2, 64–66]. A suite of 3C-derived high-throughput DNA sequencing assays have been developed, including circular chromosome conformation capture sequencing (4C-seq) [64, 67], chromosome conformation capture carbon copy (5C) [65], chromatin interaction analysis by paired-end tag sequencing (ChIA-PET) [66], enrichment of ligation products (ELP) [68] and higher resolution chromosome conformation capture sequencing (Hi-C) [2], which vary in complexity or the scale of the interactions that are captured. The initial 3C method used PCR to quantify specific ligation products between a target sequence and a small number of defined regions [1]. 4C-seq, known as the “one vs all” method, uses an inverse PCR approach to convert all chimeric molecules associated with a specific region of interest generated in the proximity ligation step into a high-throughput DNA sequencing library [67]. 5C increased the number of regions that could be captured by multiplexing PCR reactions [65], and it is also considered as the first “many vs many” approach and has been used to examine the long-range interactions of between transcription start sites and approximately 1% of the human genome [69]. ChIA-PET implements a similar approach, however uses a specific, bound protein, generally a transcription factor protein, generating a protein-centric interaction profile [31]. ELP implements a double digestion strategy to improve the enrichment of 3C products in the library and is able to generate a detailed genome-wide contact map of the yeast genome [68].

Compared to other approaches, Hi-C, also known as the genome conformation capture method [70], is the first “all vs all” method of genome-wide, 3C-derived assay to capture all interactions in the nucleus, allowing for a more complete snapshot of nuclear conformation at the global level [36]. Hi-C works through cross-linking DNA molecules in close proximity via a formaldehyde treatment, preserving the 3D interaction between two genomic regions. The cross-linked DNA is then usually fragmented using a restriction enzyme, such as the 6-bp recognition enzyme *HindIII* [30, 71] or 4 bp cutter *MboI*, *DpnII* and *Sau3AI*, and the resultant DNA, ends held in close spatial proximity by the DNA cross-links, are ligated into chimeric DNA fragments. Subsequent steps convert these chimeric DNA fragments into linear fragments to which sequencing adapters are added to create a Hi-C library. The library is then sequenced using high-throughput sequencing technology, specifically limited to Illumina paired-end (as opposed to single-end/fragment) DNA sequencing to enable the accurate identification of the two ends of the hybrid molecule [2]. In the initial development of Hi-C, the identification of Hi-C interactions was impacted by the number of spurious ligation products generated as a result of the ligation step being carried in solution allowing for greater freedom for random inter-complex ligation reactions to occur. The resolution of Hi-C interactions in these earlier approaches was also limited by the cutting frequency of a 6-base restriction enzyme, such as *HindIII *[2, 30, 72–74]. To address these issues, an in situ Hi-C protocol was developed [3], where the ligation steps were performed within the constrained space of the nuclei, reducing the chance of random ligation [75, 76]. Furthermore, in situ Hi-C used a 4-base-cutter (such as *MboI*) for digestion, increasing the cutting frequency in the genome and improving the resolution of captured interactions [3]. Using this method, the first 3D map of the human genome was constructed using the GM12878 cell line with approximately 4.9 billion interactions [3], enabling interaction resolution at the kilobase level. In recent years, the in situ Hi-C protocol has been developed further to target different technical and/or biological questions (Table 1)**.**

**Table 1:** Different Hi-C-derived methods. Optimisations indicate their modification in their protocols compared to traditional Hi-C

Hi-C flavours	Optimisations	Advantages compared to traditional Hi-C	Reference
Traditional Hi-C	–	–	[2]
In situ Hi-C	Nuclear ligation; 4-based cutter	Allow higher resolution data generation	[3]
DNase Hi-C	DNase I to digest cross-linked DNA	Improve capture efficiency, reducing digestion bias but have A compartment bias	[77]
Micro-C	Crosslinking with DSG and micrococcal nuclease to digest cross-linked DNA	Improve capture efficiency, reducing digestion bias but have A compartment bias	[78]
BL-Hi-C	HaeIII to digest cross-linked DNA, followed by a two-step ligation	Improve capture efficiency in regulatory regions, reducing random ligation events	[79]
DLO Hi-C	No labelling and pull-down step	Reduce experimental cost	[80]
tag Hi-C	Tn5-transposase tagmentation	Focus on accessible chromatin, allow only hundreds of cells as input, reduce experimental cost	[81]
Capture HiC	RNA baits to subset specific chromatin contacts	Reduce sequencing cost, focus on a subset of interactions	[82]
Capture-C/NG Capture-C/Tiled-C	Enrich the 3C library with biotinylated capture oligonucleotides	Focus on the subset of interactions while retaining maximal library complexity	[83–85]
HiChIP/PLAC-seq	Chromatin Immunoprecipitation (ChIP) to subset bound chromatin contacts	Reduce sequencing cost, focus on a subset of interactions	[86, 87]
OCEAN-C	Phenol–chloroform extraction step	Focus on accessible chromatin	[88]
HiCoP	Column purified chromatin step	Focus on accessible chromatin	[89]
Methyl-HiC	Bisulfite conversion	Allow jointly profiling of DNA methylation and 3D genome structure	[90]
Hi-C 2.0	Efficient unligated ends removal	Largely reduce the dangling end DNA products	[91]
Hi-C 3.0	Double cross-linking with FA and DSG and double digestion with *DpnII* and *DdeI*	Improve the ability to identify A/B compartments and improve the enrichment of regulatory elements in loop detection	[92]

Owing to the vast complexity of the Hi-C ligation products generated, it is often too costly to sequence samples to a sufficient depth to achieve the resolution necessary to investigate specific interactions such as promoter–enhancer interactions, leading to the development of capture Hi-C (CHi-C) [82]. CHi-C employs a sequence capture approach, using pools of probes complementary to thousands of restriction fragments, to enrich for molecules containing the region of interest from the Hi-C library. This significantly reduces the complexity of the libraries and enables a significant increase in the number of detectable interactions within specific regions without the need for ultra-deep sequencing. Therefore CHi-C, has been used in many cases to analyse specific types of long-range interactions, such as interactions linked to promoter or enhancer regions. For example, CHi-C was recently used to characterise promoter interactions in 17 human primary hematopoietic cells to demonstrate the highly cell type-specific nature of many promoter interactions even with a group of related cell types [51]. Similar to CHi-C, another series of approaches, including Capture-C [83], NG Capture-C [84] and Tiled-C [85], that focus on capturing chromatin interaction of interest have been developed. Compared to the CHi-C protocols, they enrich the 3C library with biotinylated capture oligonucleotides instead of enrich the biotinylated Hi-C library, allowing the library to retain maximal library complexity, which is important for analysing data from small cell numbers [85].

Like many other high-throughput sequencing approaches, Hi-C continues to be modified to improve the efficiency and resolution of the approach. DNase Hi-C was developed to reduce the bias introduced through the use of restriction enzymes (e.g. MboI recognises GATC), due to the uneven distribution of restriction sites throughout the genome [77, 93]. Instead, DNase Hi-C replaces the restriction enzyme digestion of cross-linked DNA with the endonuclease DNase I that has a much reduced DNA sequence specificity to reduce bias in identifying Hi-C interactions. Commercial Hi-C library preparation kit such as Omni-C kit from Dovetail Genomics [94] exploits the use of DNase and is designed specifically to overcome limitations of only capturing Hi-C interactions near restriction sites. Similar to DNase Hi-C, Micro-C uses micrococcal nuclease (MNase) digestion, enabling the generation of high-resolution contact maps at 200 bp to ~ 4 kb scale in budding yeast [78] and sub-kilobase resolution contact maps in mammalian cells [41, 95]. What’s more, BL-Hi-C uses *HaeIII*, which has higher cutting frequency in the human genome compared to other 4-base cutter like *MboI*, to conduct digestion and a two-step ligation optimisation to reduce the chance of ligating event of random DNAs, increasing the capture efficiency with active regions in the genome and reducing the probability of random ligation events [79]. In addition to increasing the capture efficiency, optimised protocols are now much more cost effective. For example, DLO Hi-C [80] avoids biotin labelling and pull-down steps, and tagHi-C [81] uses Tn5-transposase tagmentation, similar to ATAC-seq, to capture the chromatin structure with hundreds of cells.

The integration of Hi-C with other genomic applications, such as chromatin immunoprecipitation (ChIP), formaldehyde-assisted isolation of regulatory elements (FAIRE) or bisulfite treatment has also occurred. The ChIP-integrated approaches, including HiChIP and PLAC-seq, combining the in situ Hi-C with ChIP, generating a Hi-C library enriched for interactions associated with specific bound proteins [86, 87], increasing the resolution of the library while reducing the sequencing cost. Combining the phenol–chloroform extraction step from FAIRE-seq [96] with in situ Hi-C, OCEAN-C was developed to prioritise the chromatin interactions on open chromatin [88]. Similarly, integrating with an assay called column purified chromatin (CoP), which is enriched for accessible chromatin regions such as active promoters, enhancers and insulators, HiCoP was recently developed to identify chromatin contacts in regulatory regions [89]. Methyl-HiC has been developed to jointly profile the DNA methylation and 3D genome structure [90]. Recent studies have also revealed that DNA methylation is able to impact 3D genome structure via polycomb complexes, which play an important part in repressing key developmental genes [27, 97–100].

The optimisations introduced by protocols such as Micro-C largely improve the cross-linked DNA capture specificity, allowing higher resolution data to be generated with less sequencing cost. Based on these optimisations, Hi-C 2.0 and Hi-C 3.0 have been developed as the updated versions of Hi-C protocol in recent years [91, 92]. In Hi-C 3.0, the protocol uses a combination of two restriction enzymes, *DdeI* and *DpnII*, and MNase to generate short fragments, which can improve the identification of genome compartmentalisation. Additionally, Hi-C 3.0 also uses DSG as cross-linker in addition to formaldehyde to generate cross-linked DNA, improving the enrichment level of regulatory elements such as promoters and enhancers in the identified chromatin loops [92].

As the development of Hi-C approaches continue, it is essential that computational methods are standardised in order to provide consistent results that are comparable across species or cell types. In the next section, we review the current data processing methods that are used in standard Hi-C sequencing approaches.

### Prioritisation of chromatin interactions

**Fig. 2:**
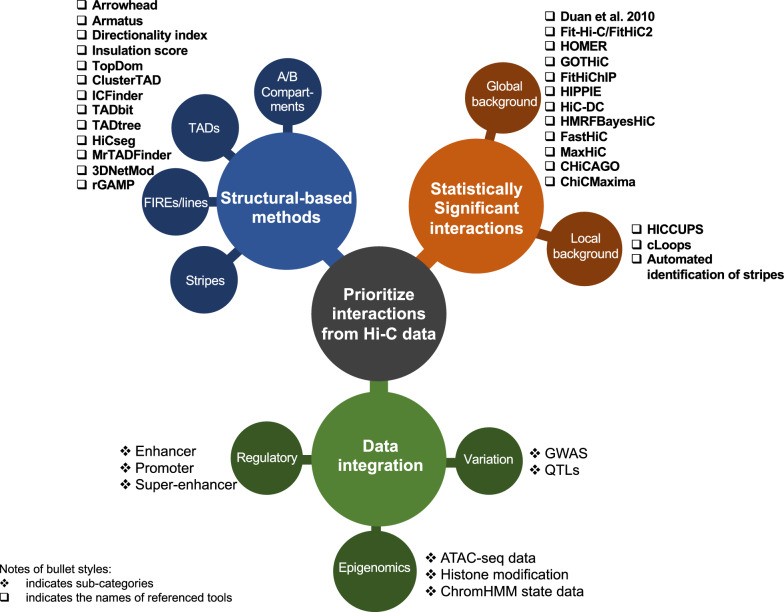
Approaches to prioritise interactions from Hi-C datasets. In this review, we categorised the approaches to identify potentially functional interactions into three ways, including significant interactions identification, structures summarisation and data integration. Referenced tools and sub-categorical analyses are marked on the figure with boxes and stars, respectively

Methodologies to extract meaningful, potentially functional information from the massive number of interactions identified through Hi-C data can be categorised into three groups: structural-based methods, detection of significant interactions and data integration (Fig. 2). The first approach is to define structures such as A/B compartments and TADs, based on the 2D interaction patterns across the genome. The second approach is to investigate only a subset of Hi-C interactions that are identified from a statistical test based on a trained model. Finally, taking advantage of the publicly available databases or the generation of epigenomics data in parallel with Hi-C data, the third approach is to prioritise interactions that are more likely to be biologically relevant through the investigation of genomic and epigenomic information. These approaches are not mutually exclusive and in many cases can be combined to address specific questions in genome organisation and gene regulation.

#### Structural-based identification methods

Methods that identify structural aspects of chromatin interactions (i.e. A/B compartments and TADs) are employed as an avenue to reduce the dimensionality of the 3D interaction patterns across the genome by clustering or summarising regions with similar patterns across the genome. The A/B compartments are commonly predicted with normalised Hi-C matrices generated using vanilla coverage (VC) [2], Knight and Ruiz’s method (KR) [101] or iterative correction and eigenvector decomposition (ICE) [102]. Normalised data are then used to calculate Pearson’s correlation and through principal component analysis (PCA), the eigenvectors of the first (or second) principal component (PC) are usually used to assign bins to A or B compartments. Current analysis toolkits, such as Juicer [103] and FAN-C [104], have optimised correlation matrix functions to identify A/B compartments from Hi-C matrices without significant taxes on memory and computational resources.

As detailed above, TADs are defined as structures with interactions that occur within TADs rather than across TADs [30]. As such, they are often identified by finding domains where contacts are enriched within the same TAD as compared to neighbouring TADs [30, 105]. Currently, there are over 20 commonly used TADs callers that have been developed using various methodologies. For instance, arrowhead [3], armatus [106], directionality index [30], insulation score [107] and TopDom [108] use their own linear scoring system, clusterTAD [109] and ICFinder [110] are based on clustering, TADbit [111], TADtree [112] and HiCseg [113] use statistical models; and MrTADFinder [114] and 3DNetMod [115] rely on network-modelling approaches [37, 116]. Although comparisons reveal low reproducibility among tools, especially in the number and mean size of identified TADs, recent reviews [37, 116] have suggested a preference for TAD callers that allow for the detection of nested TADs or overlapped TADs, such as rGMAP [117], armatus, arrowhead and TADtree.

While theoretically similar to TAD calling, frequently interacting regions (FIREs) are also commonly used to describe structural interaction characteristics. Defined as genomic regions with significant interaction profile, FIREs exhibit strong connectivity with multiple regions in the chromosome neighbourhood [73]. FIREs can be easily visualised on the Hi-C interaction map, with interacting signals appearing from both sides of the FIREs, forming a characteristic “V” shape (Fig. 1A). Unlike TADs and compartments, which exhibit a certain level of conservation across cell types (about 50 ~ 60 and 40%, respectively) [3, 30, 73, 118], FIREs appear to be cell type- and tissue-specific and are often located near key cell phenotype-defining genes. However, similar to TADs, FIREs formation seems to be dependent on the Cohesin complex, as its depletion results in decreasing interactions at FIREs [73]. They are also enriched for super-enhancers, suggesting FIREs play an important role in the dynamic gene regulation network [119, 120]. Similar to FIREs, “V” shape structural feature that is referred to as “line” structure was observed at the edge of the TADs during the exploration or loop extrusion model using simulated Hi-C data [14].

#### Methods for identification of significant chromatin interactions

In order to prioritise potentially meaningful chromatin interactions, statistical significance is assigned to Hi-C interactions by comparing them to a background model and assessing the probability of observing the experimental set of counts if the background model were the underlying method of generating observed counts. The interaction frequency generally decays with increasing linear distance, and by applying this background model meaningful interactions can be identified through a higher than normal frequency. Here we summarise the current methodologies of significant interactions identification and categorise them into two groups; global background model methods, which define a background signal model by considering the read count of any pair of interactions, and local background model methods, which account for interactions in the neighbouring areas to identify peak interactions with statistical significance (Table 2).

**Table 2:** Methods for identification of statistically significant interactions for Hi-C data

Method name	Type	Base model	Specific features	Reference
Duan et al. 2010	Global background	Binomial	Specifically designed for yeast genome	[121]
Fit-Hi-C/FitHiC2	Global background	Binomial	Spline fitting procedure, compatible with different formats	[122, 123]
HOMER	Global background	Binomial	Highly compatible with the HOMER Hi-C analysis pipeline	[124]
GOTHiC	Global background	Binomial	Use relative coverage to estimate biases	[125]
FitHiChIP	Global background	Binomial	Specifically designed for HiChIP data	[126]
HIPPIE	Global background	Negative binomial	Account for fragment length and distance biases	[72, 127]
HiC-DC	Global background	Negative binomial	Use zero-inflated model	[128]
HMRFBayesHiC	Global background	Negative binomial	Use hidden Markov random field model	[129]
FastHiC	Global background	Negative binomial	An updated version of HMRFBayesHi, with improved computing speed	[130]
MaxHiC	Global background	Negative binomial	Use ADAM algorithm, identify interactions with enrichment for regulatory elements	[131]
CHiCAGO	Global background	Negative binomial	Specifically designed for CHi-C data	[132]
ChiCMaxima	Global background	Local maxima	Specifically designed for CHi-C data, more stringent and robust when comparing biological replicates	[133]
HICCUP	Local background	Local enrichment	Robust for finding chromatin loops	[3]
cLoops	Local background	DBSCAN	Loop detection with less computational resource	[134]
Automated identification of stripes	Local background	Local enrichment	Specifically designed to identify architectural stripes	[135]

### Global background-based methods

The initial study which assigns statistical significance to Hi-C interactions is done in the yeast genome. The chromatin interactions in the yeast genome was first separated into intra-chromosomal interactions (within the same chromosome) and inter-chromosomal interactions (across two chromosomes), followed by a binomial distribution to assign confidence estimates for inter-chromosomal interactions [121]. A binning method is then used to account for the characteristic pattern of intra-chromosomal interactions, with the observed interacting probability decaying as the genomic distance increases linearly. This is then used to compute interacting probabilities for each bin separately and assigning statistical significance using the same binomial distribution as used for inter-chromosomal interactions [121]. Based on the same binomial distribution concept, Fit-Hi-C uses spline fitting procedure instead of binning, reducing the bias of artifactual stair-step pattern, allowing detection of statistically significant interactions in the mammalian genome [122]. Additionally, Fit-Hi-C also incorporates an extra refinement step using a conservative model with stringent parameters to remove outlier interactions, which can be applied iteratively, to achieve a more accurate empirical null model. However, Fit-Hi-C was initially limited by only allowing bin sizes larger than 5 kb to compute significance due to the heavy memory usage when dealing with higher resolution data. However this has been improved with recent updates [123], and is now able to handle data with high resolution (bin sizes from 1 to 5 kb). Another important new feature is that it is now accepting multiple input formats so that it is compatible with different Hi-C analysis pipelines. Another similar tool is included in the Homer toolkit [124], which accounts for biases such as sequencing depths, linear distance between regions, GC bias and chromatin compaction to establish a background model to estimate the expected interaction count between any two regions, followed by the use of a cumulative binomial distribution to assign significance to interactions. GOTHiC [125] also uses relative coverage of two interacting regions to estimate both known and unknown biases, followed by a cumulative binomial distribution to build the background model to identify significant interactions.

The Negative Binomial distribution is commonly utilised in the analysis of count-based data, including popular RNA-seq analysis tools such as edgeR [136] and DEseq2 [137], and has been implemented in a number of Hi-C programs such as HIPPIE [72, 127]. This method uses a negative binomial model to estimate the statistical significance of the interactions in one fragment region (< 2 Mb) while accounting for restriction fragment length bias and interacting probability distance bias simultaneously. However, negative binomial models can be confounded by many bins with zero counts [128] and a number of programs have developed approaches to account for “zero-inflated” observations. HiC-DC, for example, uses a hurdle negative binomial regression model to identify significant interactions [128], modelling the probability of non-zero counts and the rate of observed counts as separate components of the model.

While physical interactions between loci found in close linear proximity are likely to be more prevalent in Hi-C datasets, a known bias in Hi-C libraries is the correlation between two nearby restriction fragments brought about by ligation events. Ligation events can be the result of bias or random collision events between restriction fragments during library preparation, so with high coverage sequencing, false signals can impact the identification of significant interactions [72]. To tackle this problem, HMRFBayesHiC uses a negative binomial distribution to model observed interactions [72], followed by a hidden Markov random field model to account for the correlation between restriction fragments, and to model interaction probabilities [129]. This implementation required significant resources to run, leading to the development of FastHiC [130], which enables higher accuracy of interaction identification and faster performance. Recently, another tool called MaxHiC also based on negative binomial distribution was developed [131]. Compared to other tools, all parameters of the background model in MaxHiC are established by using the ADAM algorithm [138] to maximise the logarithm of likelihood of the observed Hi-C interactions. Significant interactions identified by MaxHiC were shown to outperform tools such as Fit-Hi-C/FitHiC2 and GOTHiC in identifying significant interactions enriched between known regulatory regions [131].

Compared to traditional Hi-C protocols, Capture Hi-C (CHi-C) requires different analytic methods due to the extra bias driven by the enrichment step in the protocol. Capture libraries can be regarded as a subset of the original Hi-C library, meaning the interaction matrix of CHi-C is asymmetric, and interestingly not accounted for in traditional normalisation methods [82, 132]. Because of this, many analysis approaches are specifically designed for CHi-C data analysis. CHiCAGO (Capture Hi-C Analysis of Genomic Organisation) was developed to account for biases from the CHi-C protocol and identify significant interactions [132], using a negative binomial distribution to model the background local profile and an additional Poisson random variable to model technical artefacts [132]. CHiCAGO uses the implicit normalisation method ICE [102] and multiple testing stages based on p-value weighting [139] to carefully identify significant interactions from each CHi-C dataset [132]. Another CHi-C-specific tool called ChiCMaxima was developed to identify significant interactions by defining them as local maxima after using loess smoothing on bait-specific interactions [133]. Compared to CHiCAGO, ChiCMaxima’s approach is more stringent and exhibits a more robust performance when comparing biological replicates [133]. As well as being applicable to conventional HiC data, MaxHiC is also able to identify significant interactions in CHi-C data [131] and offers robust performance to identify regulatory areas compared to CHi-C-specific tools including CHiCAGO [131].

Like the other capture approaches, HiChIP cannot use traditional (Hi-C-specific) interaction callers (e.g. Fit-Hi-C or GOTHiC) due to the inherent biases associated with an enrichment with specific immunoprecipitation targets [86]. Hichipper was developed to firstly identify ChIP peaks while accounting for the read density bias in restriction fragments, enabling a more accurate identification of interactions from HiChIP dataset [140]. While hichipper does not implement any function to identify significant interactions, FitHiChIP was developed to account for non-uniform coverage bias and distance bias in restriction fragments using a regression model, together with 1D peak information in a spline fitting procedure to accurately identify significant interactions from HiChIP data [126].

### Local background-based methods

Chromatin looping structures can be regarded as the basic unit of 3D genomic architecture and play an important role in the regulatory process, by bringing distal promoter and enhancer elements together or excluding enhancers from the looping domain [15–17]. Chromatin loops from Hi-C data were first defined by searching for the strongest “pixel” on a normalised Hi-C contact map (Fig. 1A). Different from the global background models used by methods like Fit-Hi-C and MaxHiC, using a local background model to compare all pixels in a neighbouring area is able to detect pixels with the strongest signals as the anchor points of chromatin loops [3]. A searching algorithm named Hi-C Computational Unbiased Peak Search (HICCUPS) was therefore developed to rigorously search for these pixels based on the local enrichment in the pixel neighbourhood, followed by hypothesis testing with Poisson statistics, enabling the identification of chromatin loops from Hi-C data [3]. Somewhat similar to TADs, published information on chromatin loops demonstrates structural conservation between a number of human cell lines (~ 55–75% similarity), and between human and mouse (about 50% similarity), suggesting conserved loops may serve as a basic functional unit for the genome [3]. However, loop detection using HICCUPS requires high-resolution data with extremely high sequencing depth. For example, almost 5 billion unique interactions were required by HICCUPS to identify 10,000 unique loops in the GM12878 cell line [3]. This limitation can potentially be addressed by the current development of deep learning approaches, such as DeepHiC [141] using generative adversarial networks, as well as HiCPlus [142] and HiCNN [143] which use deep convolutional neural networks. Such methods can be used to increase the resolution of Hi-C data to achieve necessary resolution so that chromatin loops can be identified, or to improve loop detection accuracy [141, 142].

Hardware requirements to identify loops in high-resolution data is also extremely restrictive with HICCUPS requiring specific architectures (i.e. NVIDIA GPUs) to identify looping patterns. However this has been addressed recently with the HICCUPS algorithm being reimplemented in the cooltools package (https://github.com/mirnylab/cooltools), allowing HICCUPS to be run on a regular server or compute cluster [95]. Alternatively, an approach called cLoops was implemented which identifies peak interactions from chromatin contact map [134]. cLoops initiates loop detection by finding candidate loops via an unsupervised clustering algorithm, Density-Based Spatial Clustering of Applications with Noise (DBSCAN) [144], which enables computing statistical significance of interactions with less amount of input and reduced computational resources. Candidate loops are then compared with a permuted background model, based on the interaction decay over linear distance, to estimate statistical significance.

Further investigation in high-resolution Hi-C data (< = 10 kb), another local background model method was developed to identify architectural stripe structures rather than loops [135]. The stripe structure is similar to FIRE, where a genomic region contacts other regions of the entire domain with high interacting frequency [135]. Its identification algorithm *Automated identification of stripes* computes the pixel-specific enrichment relative to its local neighbourhood, then performs Poisson statistics to test if the signal is statistically significant [135]. It was further shown that stripe anchors highly correspond to loop anchors, and stripes appear to be relevant with enhancer activity [135, 145].

### Potentially functional interaction identification via data integration

While variation in gene-coding regions can lead to significant alterations in one gene or abnormalities across a region in the genome, causing mendelian diseases such as chronic granulomatous disease [146], cystic fibrosis [147] and Fanconi’s anaemia [148], the fundamental motivation for identifying interacting regions across a genome is to establish how non-coding regions of the genome impact gene expression [1, 149, 150]. However potentially functionally relevant interactions, whether this be chromatin interactions between gene promoters and enhancers or transcription factor binding mechanisms, are often established in a cell type-specific manner [71, 82]. By integrating Hi-C interactions with local or publicly available genomic, transcriptomic and epigenomic datasets, such as regulatory elements, gene expression, genetic variation and quantitative trait loci (QTL) information, potentially functional interactions can be prioritised.

Potentially functional Hi-C interactions can be identified by integration with transcriptomics and enhancer data. Promoter–enhancer interactions (PEI), promoter–promoter interactions (PPI) or enhancer–enhancer interactions (EEI), where distal promoters or enhancers are brought into close proximity by chromatin contacts to form complex contact, are three widely accepted potentially functional Hi-C interaction types to be studied [51, 69, 151–157]. These interaction categories are often identified by finding overlaps of promoter or enhancer signals separately at each anchor of a Hi-C interaction [51, 155, 158]. However, when identifying PEI or PPI from Hi-C data for a specific cell type, the gene expression profile of such cell type should be considered to determine which promoters are active given that promoter interactions are shown highly cell-type specific [51].

Similar to promoters of expressed genes, active enhancers of a specific cell type are necessary to identify potentially functional PEI or EEI for a specific cell type. Expressed enhancers (eRNAs) or experimentally verified enhancers of different human cell types and tissues are available in publicly available projects and databases such as FANTOM5 project [159], the NIH Roadmap Epigenomics project [160], the EU Blueprint project [161], ENCODE [162, 163] and ENdb [164]. Additionally, previous studies also used cell type-specific histone markers ChIP-seq data, such as H3K27ac and H3K4me1, or integrated chromHMM chromatin state information predicted from a variety of epigenomic sequencing information [165, 166] to indicate the activity of an enhancer in a specific cell type [51, 155, 158, 167, 168]. In addition to using Hi-C data, there are numerous methods that have been developed to predict potentially functional interactions based on histone marker signals [169], gene expression and methylation data [170], ATAC-seq data [171], DNase-seq data [172] or even DNA sequence alone [173]. These types of methods have been comprehensively reviewed in a recent review study [174].

Besides promoters and enhancers, Super-enhancers (SEs) are another major regulatory element that is crucial to the identification of potentially functional interactions. SEs are defined as a clustered region of enhancers exhibiting significantly higher levels of active enhancer marks and an enrichment with transcription factor binding sites (TFBS) [175]. These regions act as “regulatory hubs”, which are higher-order complexes consisting of interactions between multiple enhancers and promoters at individual alleles [152, 176, 177]. The formation of these regulatory hubs are proposed to be the consequence of the high level of TF and co-factor localisation to the SE interacting to form a biomolecular condensate by a phase separation model [178–183]. Identified Hi-C interactions with linkages to SE have been shown to be potentially functional by mediating multiple gene expression regulations three-dimensionally, or being essential for cell identity and development [50, 184–189]. SE can be identified from H3K27ac ChIP-seq using the ROSE algorithm [186], and currently SE information can be easily accessible from databases such as AnimalTFDB [190], PlantTFDB [191], GTRD [192], SEdb [193], dbSUPER [194] and SEA [195, 196], allowing cell-type regulatory hubs to identified and linked to phenotypic traits and/or disease.

In genome-wide association studies (GWAS), almost 90% of the identified genetic single-nucleotide polymorphisms (SNPs) associated with phenotypic traits are located in non-coding regions such as gene desert, which are areas lacking protein-coding genes, hence making the interpretation of the functions of such variants much more challenging than the ones located within or nearby protein-coding genes [197–199]. Hi-C data have been proved to be useful in many studies for addressing this issue by forming linkages between diseases-associated variants and genes using long-range chromatin interactions. For examples, interactions between gene promoters and variation-located long coding RNAs (lncRNA), where GWAS SNPs can impact the expression of the target genes by affecting the binding of TF binding to the lncRNA [200]; direct interactions between SNPs and multiple genes, exhibiting co-regulation function of the SNPs [201]; interaction networks based on a SNP, bringing gene promoter, TF binding site and active enhancer region together by chromatin interactions to affect gene expression [202]. Variants may also impact gene-coding regions over large distances meaning that target genes of the variations are not necessarily their closest proximal gene [71, 203]. Currently, databases such as GWAS catalog [204], ImmunoBase [205], GWAS Central [206], GWAS ALTAS [207] and GWASdb [208] contain information of the level of genetic association of each variant to specific diseases, which are invaluable data to be integrated in a high-dimensional interaction dataset.

Tissue-specific quantitative trait loci (QTLs) are identified as the possession of variants that can significantly impact the level of quantitative trait [209], such as expression QTLs (eQTLs) that affect the expression level of the target genes [210], histone QTLs (hQTLs) that affect histone modifications [211, 212], methylation QTLs (meQTLs) that impact DNA methylations [213, 214] and ATAC-QTL that affect the accessibility of the corresponding areas [215]. In recent QTL studies, QTLs are found to affect their target regions by the long-range chromatin interactions between them observed from Hi-C data. For example, Greenwald et al*.* has recently used pancreatic islet-specific data to investigate the risk gene loci of type 2 diabetes (T2D) [216]. In their work they combined gene and enhancers interaction maps generated from Hi-C data, together with variant and gene expression linkage data, provided by tissue-specific eQTL analysis, to establish an enhancer network for T2D risk loci. In support of genetic variation at enhancers influencing transcriptional regulation, Yu et al*.* used HiC data to demonstrate that eQTLs tend to be in close spatial proximity with their target genes [217]. Additionally, a recent multi-tissues integration analysis between eQTLs and Hi-C interactions revealed the close proximity between eQTLs and their target genes, indicating that eQTLs regulate the expression of their target genes through chromatin contacts [217]. Therefore, with publicly available QTL databases such as the GTEx project [210], seeQTL [218], Haploreg [219], Blood eQTL browser [220], Pancan-meQTL [221] and QTLbase [222], the linkages between such QTLs and their target genes or regions can be used to infer potentially functional Hi-C interactions.

### Future prospects

The investigation of 3D chromosome structure can provide novel insights into the complex regulatory network in the genome. The development of Hi-C and its derived protocols have facilitated the studies of the 3D genome structure, generating numerous high-quality datasets. However, due to the complexity of the Hi-C library preparation and analysis, the biologically meaningful, small-scale interactions may still lack sufficient signals, hindering the detection and interpretation of 3D interactions. The approaches that we presented in this review all aim to reduce the complexity of 3D interaction data, narrowing down information based on structure, statistical inference and additional lines of experimental evidence (i.e. cell type-specific epigenomic data).

Incremental development of Hi-C calling applications (chromatin loops, TADs, etc.) has continued with a focus on correcting biases introduced by library preparation and sequencing. As more and more sequencing data are deposited on open-access data repositories such as NCBI Short Read Archive (SRA) [223] and European Nucleotide Archive (ENA) [224], it has allowed the development of novel Machine Learning models trained on known interactions to identify novel patterns when applying these models to new datasets. Incorporation of publicly available cell type/tissue-specific epigenomics data into these machine learning models of chromatin interactions will allow for more accurate predictions on the molecular mechanisms by which diseases-associated genetic acts. In the future, such models of 3D interactions can potentially be used as markers for disease screening and used for personalised medicine development.

Although the development in protocol efficiency, parallel algorithmic improvements are likely to improve current approaches for identifying 3D interactions. Additional imaging technologies such as real-time signal fluorescence in situ hybridisation and advanced imaging approaches such as STORM imaging have been used to visualise the nuclear organisation in living cells and leading to the identification of clusters of clutch domains that are thought to correspond to TAD [7, 225]. Lastly the ability to engineer specific mutations in DNA through genome editing technology such as the CRISPR–Cas9 system [226, 227], means that future experiments using Hi-C and 3D imaging in-parallel with genetically modification of genomes will vastly improve our understanding of how variation may impact genomic structure, and the regulations of gene expression.

## Conclusion

In this review, we first introduced the three-dimensional chromosome architecture in different scales, followed by presenting the chromosome conformation capture assays, with a focus on Hi-C and its variations, which are the state-of-the-art methods for investigating the 3D genome structure. Lastly, we comprehensively reviewed methodologies that are developed to reduce the complexity of 3D physical interactions identified from Hi-C datasets to detect potentially functional interactions. We also categorised the methods into three types, including structural-based detection methods, significant chromatin interactions identification methods and data integration methods. Taken together, by utilising these methods carefully, we are able to detect physical interactions with biological meaning and impact from complicated Hi-C dataset, which may serve a purpose in diagnosis and precision medicine.

## Data Availability

Not applicable.

## Abbreviations

CTCF: CCCTC-binding protein
TAD: Topologically associated domain
FIRE: Frequently interacting region
NADs: Nucleolus-associated domains
3D-FISH: 3D fluorescence in situ hybridisation
3C: Chromosome conformation capture
4C-seq: Circular chromosome conformation capture sequencing
5C: Chromosome conformation capture carbon copy
ChIA-PET: Chromatin interaction analysis by paired-end tag sequencing
Hi-C: Higher-resolution chromosome conformation capture sequencing
CHi-C: Capture Hi-C
PEI: Promoter–enhancer interactions
PPI: Promoter–promoter interactions
EEI: Enhancer–enhancer interactions
SE: Super-enhancers
TFBS: Transcription factor binding sites
lncRNA: Long coding RNAs

## References

[1] Dekker J, Rippe K, Dekker M, Kleckner N (2002). Capturing chromosome conformation. Science.

[2] Lieberman-Aiden E, van Berkum NL, Williams L, Imakaev M, Ragoczy T, Telling A (2009). Comprehensive mapping of long-range interactions reveals folding principles of the human genome. Science.

[3] Rao SSP, Huntley MH, Durand NC, Stamenova EK, Bochkov ID, Robinson JT (2014). A 3D map of the human genome at kilobase resolution reveals principles of chromatin looping. Cell.

[4] Taberlay PC, Achinger-Kawecka J, Lun ATL, Buske FA, Sabir K, Gould CM (2016). Three-dimensional disorganization of the cancer genome occurs coincident with long-range genetic and epigenetic alterations. Genome Res.

[5] Anania C, Lupiáñez DG (2020). Order and disorder: abnormal 3D chromatin organization in human disease. Brief Funct Genomics.

[6] Liu N, Sadlon T, Wong YY, Pederson SM, Breen J (2020). 3DFAACTS-SNP: Using regulatory T cell-specific epigenomics data to uncover candidate mechanisms of Type-1 Diabetes (T1D) risk. bioRxiv..

[7] Lakadamyali M, Cosma MP (2020). Visualizing the genome in high resolution challenges our textbook understanding. Nat Methods Nature Publishing Group.

[8] Rowley MJ, Corces VG (2018). Organizational principles of 3D genome architecture. Nat Rev Genet.

[9] Bonev B, Cavalli G (2016). Organization and function of the 3D genome. Nat Rev Genet.

[10] Maass PG, Barutcu AR, Rinn JL (2019). Interchromosomal interactions: a genomic love story of kissing chromosomes. J Cell Biol.

[11] Davidson IF, Bauer B, Goetz D, Tang W, Wutz G, Peters J-M (2019). DNA loop extrusion by human cohesin. Science.

[12] Nasmyth K (2001). Disseminating the genome: joining, resolving, and separating sister chromatids during mitosis and meiosis. Annu Rev Genet.

[13] Sanborn AL, Rao SSP, Huang SC, Durand NC, Huntley MH, Jewett AI (2015). Chromatin extrusion explains key features of loop and domain formation in wild-type and engineered genomes. PNAS.

[14] Fudenberg G, Imakaev M, Lu C, Goloborodko A, Abdennur N, Mirny LA (2016). Formation of chromosomal domains by loop extrusion. Cell Rep.

[15] Kadauke S, Blobel GA (2009). Chromatin loops in gene regulation. Biochim Biophys Acta.

[16] Guo Y, Xu Q, Canzio D, Shou J, Li J, Gorkin DU (2015). CRISPR inversion of CTCF sites alters genome topology and enhancer/promoter function. Cell.

[17] Krijger PHL, de Laat W (2016). Regulation of disease-associated gene expression in the 3D genome. Nat Rev Mol Cell Biol.

[18] Splinter E, Heath H, Kooren J, Palstra R-J, Klous P, Grosveld F (2006). CTCF mediates long-range chromatin looping and local histone modification in the beta-globin locus. Genes Dev.

[19] Rubio ED, Reiss DJ, Welcsh PL, Disteche CM, Filippova GN, Baliga NS (2008). CTCF physically links cohesin to chromatin. Proc Natl Acad Sci U S A.

[20] Zuin J, Dixon JR, van der Reijden MIJA, Ye Z, Kolovos P, Brouwer RWW (2014). Cohesin and CTCF differentially affect chromatin architecture and gene expression in human cells. Proc Natl Acad Sci U S A.

[21] Banigan EJ, van den Berg AA, Brandão HB, Marko JF, Mirny LA (2020). Chromosome organization by one-sided and two-sided loop extrusion. Elife.

[22] Banigan EJ, Mirny LA (2020). Loop extrusion: theory meets single-molecule experiments. Curr Opin Cell Biol.

[23] Drissen R, Palstra R-J, Gillemans N, Splinter E, Grosveld F, Philipsen S (2004). The active spatial organization of the beta-globin locus requires the transcription factor EKLF. Genes Dev.

[24] Vakoc CR, Letting DL, Gheldof N, Sawado T, Bender MA, Groudine M (2005). Proximity among distant regulatory elements at the beta-globin locus requires GATA-1 and FOG-1. Mol Cell.

[25] Deng W, Lee J, Wang H, Miller J, Reik A, Gregory PD (2012). Controlling long-range genomic interactions at a native locus by targeted tethering of a looping factor. Cell.

[26] Apostolou E, Ferrari F, Walsh RM, Bar-Nur O, Stadtfeld M, Cheloufi S (2013). Genome-wide chromatin interactions of the Nanog locus in pluripotency, differentiation, and reprogramming. Cell Stem Cell.

[27] Denholtz M, Bonora G, Chronis C, Splinter E, de Laat W, Ernst J (2013). Long-range chromatin contacts in embryonic stem cells reveal a role for pluripotency factors and polycomb proteins in genome organization. Cell Stem Cell.

[28] Weintraub AS, Li CH, Zamudio AV, Sigova AA, Hannett NM, Day DS (2017). YY1 Is a structural regulator of enhancer-promoter loops. Cell.

[29] Robinson JT, Turner D, Durand NC, Thorvaldsdóttir H, Mesirov JP, Aiden EL (2018). Juicebox.js provides a cloud-based visualization system for Hi-C data. Cell Syst.

[30] Dixon JR, Selvaraj S, Yue F, Kim A, Li Y, Shen Y (2012). Topological domains in mammalian genomes identified by analysis of chromatin interactions. Nature.

[31] Tang Z, Luo O, Li X, Zheng M, Zhu J, Szalaj P (2015). CTCF-mediated human 3D genome architecture reveals chromatin topology for transcription. Cell.

[32] de Wit E, Vos ESM, Holwerda SJB, Valdes-Quezada C, Verstegen MJAM, Teunissen H (2015). CTCF binding polarity determines chromatin looping. Mol Cell.

[33] Beagan JA, Phillips-Cremins JE (2020). On the existence and functionality of topologically associating domains. Nat Genet.

[34] Szabo Q, Bantignies F, Cavalli G (2019). Principles of genome folding into topologically associating domains. Sci Adv.

[35] de Wit E (2019). TADs as the caller calls them. J Mol Biol.

[36] Rocha PP, Raviram R, Bonneau R, Skok JA (2015). Breaking TADs: insights into hierarchical genome organization. Epigenomics.

[37] Zufferey M, Tavernari D, Oricchio E, Ciriello G (2018). Comparison of computational methods for the identification of topologically associating domains. Genome Biol.

[38] Vietri Rudan M, Barrington C, Henderson S, Ernst C, Odom DT, Tanay A (2015). Comparative Hi-C reveals that CTCF underlies evolution of chromosomal domain architecture. Cell Rep.

[39] Rowley MJ, Nichols MH, Lyu X, Ando-Kuri M, Rivera ISM, Hermetz K (2017). Evolutionarily conserved principles predict 3D chromatin organization. Mol Cell.

[40] Llères D, Moindrot B, Pathak R, Piras V, Matelot M, Pignard B (2019). CTCF modulates allele-specific sub-TAD organization and imprinted gene activity at the mouse Dlk1-Dio3 and Igf2-H19 domains. Genome Biol.

[41] Hsieh T-HS, Cattoglio C, Slobodyanyuk E, Hansen AS, Rando OJ, Tjian R (2020). Resolving the 3D landscape of transcription-linked mammalian chromatin folding. Mol Cell.

[42] Giorgetti L, Lajoie BR, Carter AC, Attia M, Zhan Y, Xu J (2016). Structural organization of the inactive X chromosome in the mouse. Nature.

[43] Wang Q, Sun Q, Czajkowsky DM, Shao Z (2018). Sub-kb Hi-C in D. melanogaster reveals conserved characteristics of TADs between insect and mammalian cells. Nat Commun.

[44] Shen Y, Yue F, McCleary DF, Ye Z, Edsall L, Kuan S (2012). A map of the cis-regulatory sequences in the mouse genome. Nature.

[45] Nora EP, Dekker J, Heard E (2013). Segmental folding of chromosomes: a basis for structural and regulatory chromosomal neighborhoods?. BioEssays.

[46] Symmons O, Uslu VV, Tsujimura T, Ruf S, Nassari S, Schwarzer W (2014). Functional and topological characteristics of mammalian regulatory domains. Genome Res.

[47] Lupiáñez DG, Kraft K, Heinrich V, Krawitz P, Brancati F, Klopocki E (2015). Disruptions of topological chromatin domains cause pathogenic rewiring of gene-enhancer interactions. Cell.

[48] Smith EM, Lajoie BR, Jain G, Dekker J (2016). Invariant TAD boundaries constrain cell-type-specific looping interactions between promoters and distal elements around the CFTR locus. Am J Hum Genet.

[49] Hnisz D, Weintraub AS, Day DS, Valton A-L, Bak RO, Li CH (2016). Activation of proto-oncogenes by disruption of chromosome neighborhoods. Science.

[50] Huang J, Li K, Cai W, Liu X, Zhang Y, Orkin SH (2018). Dissecting super-enhancer hierarchy based on chromatin interactions. Nat Commun.

[51] Javierre BM, Burren OS, Wilder SP, Kreuzhuber R, Hill SM, Sewitz S (2016). Lineage-specific genome architecture links enhancers and non-coding disease variants to target gene promoters. Cell.

[52] Montavon T, Soshnikova N, Mascrez B, Joye E, Thevenet L, Splinter E (2011). A regulatory archipelago controls Hox genes transcription in digits. Cell.

[53] de Laat W, Klous P, Kooren J, Noordermeer D, Palstra R (2008). Chapter 5 three-dimensional organization of gene expression in erythroid cells. Red Cell Dev.

[54] Tolhuis B, Palstra RJ, Splinter E, Grosveld F, de Laat W (2002). Looping and interaction between hypersensitive sites in the active beta-globin locus. Mol Cell.

[55] Palstra R-J, Tolhuis B, Splinter E, Nijmeijer R, Grosveld F, de Laat W (2003). The β-globin nuclear compartment in development and erythroid differentiation. Nat Genet.

[56] Noordermeer D, de Laat W (2008). Joining the loops: beta-globin gene regulation. IUBMB Life.

[57] Wang KC, Yang YW, Liu B, Sanyal A, Corces-Zimmerman R, Chen Y (2011). A long noncoding RNA maintains active chromatin to coordinate homeotic gene expression. Nature.

[58] Kim YJ, Cecchini KR, Kim TH (2011). Conserved, developmentally regulated mechanism couples chromosomal looping and heterochromatin barrier activity at the homeobox gene A locus. Proc Natl Acad Sci U S A.

[59] Noordermeer D, Leleu M, Splinter E, Rougemont J, De Laat W, Duboule D (2011). The dynamic architecture of Hox gene clusters. Science.

[60] Noordermeer D, Leleu M, Schorderet P, Joye E, Chabaud F, Duboule D (2014). Temporal dynamics and developmental memory of 3D chromatin architecture at Hox gene loci. Elife.

[61] Di Giammartino DC, Polyzos A, Apostolou E (2020). Transcription factors: building hubs in the 3D space. Cell Cycle.

[62] Rickels R, Shilatifard A (2018). Enhancer Logic and Mechanics in Development and Disease. Trends Cell Biol.

[63] Smith E, Shilatifard A (2014). Enhancer biology and enhanceropathies. Nat Struct Mol Biol.

[64] Simonis M, Klous P, Splinter E, Moshkin Y, Willemsen R, de Wit E (2006). Nuclear organization of active and inactive chromatin domains uncovered by chromosome conformation capture-on-chip (4C). Nat Genet.

[65] Dostie J, Richmond TA, Arnaout RA, Selzer RR, Lee WL, Honan TA (2006). Chromosome Conformation Capture Carbon Copy (5C): a massively parallel solution for mapping interactions between genomic elements. Genome Res.

[66] Fullwood MJ, Liu MH, Pan YF, Liu J, Xu H, Mohamed YB (2009). An oestrogen-receptor-alpha-bound human chromatin interactome. Nature.

[67] Zhao Z, Tavoosidana G, Sjölinder M, Göndör A, Mariano P, Wang S (2006). Circular chromosome conformation capture (4C) uncovers extensive networks of epigenetically regulated intra- and interchromosomal interactions. Nat Genet.

[68] Tanizawa H, Iwasaki O, Tanaka A, Capizzi JR, Wickramasinghe P, Lee M (2010). Mapping of long-range associations throughout the fission yeast genome reveals global genome organization linked to transcriptional regulation. Nucleic Acids Res.

[69] Sanyal A, Lajoie BR, Jain G, Dekker J (2012). The long-range interaction landscape of gene promoters. Nature.

[70] Rodley CDM, Bertels F, Jones B, O’Sullivan JM (2009). Global identification of yeast chromosome interactions using Genome conformation capture. Fungal Genet Biol.

[71] Martin P, McGovern A, Orozco G, Duffus K, Yarwood A, Schoenfelder S (2015). Capture Hi-C reveals novel candidate genes and complex long-range interactions with related autoimmune risk loci. Nat Commun.

[72] Jin F, Li Y, Dixon JR, Selvaraj S, Ye Z, Lee AY (2013). A high-resolution map of the three-dimensional chromatin interactome in human cells. Nature.

[73] Schmitt AD, Hu M, Jung I, Xu Z, Qiu Y, Tan CL (2016). A Compendium of chromatin contact maps reveals spatially active regions in the human genome. Cell Rep.

[74] Barutcu AR, Hong D, Lajoie BR, McCord RP, van Wijnen AJ, Lian JB (2016). RUNX1 contributes to higher-order chromatin organization and gene regulation in breast cancer cells. Biochim Biophys Acta.

[75] van de Werken HJG, Landan G, Holwerda SJB, Hoichman M, Klous P, Chachik R (2012). Robust 4C-seq data analysis to screen for regulatory DNA interactions. Nat Methods.

[76] Nagano T, Lubling Y, Yaffe E, Wingett SW, Dean W, Tanay A (2015). Single-cell Hi-C for genome-wide detection of chromatin interactions that occur simultaneously in a single cell. Nat Protoc.

[77] Ma W, Ay F, Lee C, Gulsoy G, Deng X, Cook S (2015). Fine-scale chromatin interaction maps reveal the cis-regulatory landscape of human lincRNA genes. Nat Methods.

[78] Hsieh THS, Weiner A, Lajoie B, Dekker J, Friedman N, Rando OJ (2015). Mapping nucleosome resolution chromosome folding in yeast by micro-C. Cell.

[79] Liang Z, Li G, Wang Z, Djekidel MN, Li Y, Qian M-P (2017). BL-Hi-C is an efficient and sensitive approach for capturing structural and regulatory chromatin interactions. Nat Commun.

[80] Lin D, Hong P, Zhang S, Xu W, Jamal M, Yan K (2018). Digestion-ligation-only Hi-C is an efficient and cost-effective method for chromosome conformation capture. Nat Genet.

[81] Zhang C, Xu Z, Yang S, Sun G, Jia L, Zheng Z (2020). tagHi-C reveals 3D chromatin architecture dynamics during mouse hematopoiesis. Cell Rep.

[82] Mifsud B, Tavares-Cadete F, Young AN, Sugar R, Schoenfelder S, Ferreira L (2015). Mapping long-range promoter contacts in human cells with high-resolution capture Hi-C. Nat Genet.

[83] Hughes JR, Roberts N, McGowan S, Hay D, Giannoulatou E, Lynch M (2014). Analysis of hundreds of cis-regulatory landscapes at high resolution in a single, high-throughput experiment. Nat Genet.

[84] Davies JOJ, Telenius JM, McGowan SJ, Roberts NA, Taylor S, Higgs DR (2016). Multiplexed analysis of chromosome conformation at vastly improved sensitivity. Nat Methods.

[85] Oudelaar AM, Beagrie RA, Gosden M, de Ornellas S, Georgiades E, Kerry J (2020). Dynamics of the 4D genome during in vivo lineage specification and differentiation. Nat Commun.

[86] Mumbach MR, Rubin AJ, Flynn RA, Dai C, Khavari PA, Greenleaf WJ (2016). HiChIP: efficient and sensitive analysis of protein-directed genome architecture. Nat Methods.

[87] Fang R, Yu M, Li G, Chee S, Liu T, Schmitt AD (2016). Mapping of long-range chromatin interactions by proximity ligation-assisted ChIP-seq. Cell Res.

[88] Li T, Jia L, Cao Y, Chen Q, Li C (2018). OCEAN-C: mapping hubs of open chromatin interactions across the genome reveals gene regulatory networks. Genome Biol.

[89] Zhang Y, Li Z, Bian S, Zhao H, Feng D, Chen Y (2020). HiCoP, a simple and robust method for detecting interactions of regulatory regions. Epigenetics Chromatin.

[90] Li G, Liu Y, Zhang Y, Kubo N, Yu M, Fang R (2019). Joint profiling of DNA methylation and chromatin architecture in single cells. Nat Methods.

[91] Belaghzal H, Dekker J, Gibcus JH (2017). Hi-C 2.0: an optimized Hi-C procedure for high-resolution genome-wide mapping of chromosome conformation. Methods.

[92] Oksuz BA, Yang L, Abraham S, Venev SV (2020). Systematic evaluation of chromosome conformation capture assays. bioRxiv.

[93] Ramani V, Cusanovich DA, Hause RJ, Ma W, Qiu R, Deng X (2016). Mapping 3D genome architecture through in situ DNase Hi-C. Nat Protoc.

[94] Putnam NH, O’Connell BL, Stites JC, Rice BJ, Blanchette M, Calef R (2016). Chromosome-scale shotgun assembly using an in vitro method for long-range linkage. Genome Res.

[95] Krietenstein N, Abraham S, Venev SV, Abdennur N, Gibcus J, Hsieh T-HS (2020). Ultrastructural details of mammalian chromosome architecture. Mol Cell.

[96] Simon JM, Giresi PG, Davis IJ, Lieb JD (2012). Using formaldehyde-assisted isolation of regulatory elements (FAIRE) to isolate active regulatory DNA. Nat Protoc.

[97] Schoenfelder S, Sugar R, Dimond A, Javierre B-M, Armstrong H, Mifsud B (2015). Polycomb repressive complex PRC1 spatially constrains the mouse embryonic stem cell genome. Nat Genet.

[98] Vieux-Rochas M, Fabre PJ, Leleu M, Duboule D, Noordermeer D (2015). Clustering of mammalian Hox genes with other H3K27me3 targets within an active nuclear domain. Proc Natl Acad Sci U S A.

[99] Joshi O, Wang S-Y, Kuznetsova T, Atlasi Y, Peng T, Fabre PJ (2015). Dynamic reorganization of extremely long-range promoter-promoter interactions between two states of pluripotency. Cell Stem Cell.

[100] McLaughlin K, Flyamer IM, Thomson JP, Mjoseng HK, Shukla R, Williamson I (2019). DNA methylation directs polycomb-dependent 3D genome re-organization in naive pluripotency. Cell Rep.

[101] Knight PA, Ruiz D (2013). A fast algorithm for matrix balancing. IMA J Numer Anal.

[102] Imakaev M, Fudenberg G, McCord RP, Naumova N, Goloborodko A, Lajoie BR (2012). Iterative correction of Hi-C data reveals hallmarks of chromosome organization. Nat Methods.

[103] Durand NC, Shamim MS, Machol I, Rao SSP, Huntley MH, Lander ES (2016). Juicer provides a one-click system for analyzing loop-resolution Hi-C experiments. Cell Syst.

[104] Kruse K, Hug CB, Vaquerizas JM (2020). FAN-C: a feature-rich framework for the analysis and visualisation of C data. bioRxiv.

[105] Chang L-H, Ghosh S, Noordermeer D (2020). TADs and their borders: free movement or building a wall?. J Mol Biol.

[106] Filippova D, Patro R, Duggal G, Kingsford C (2014). Identification of alternative topological domains in chromatin. Algorithms Mol Biol.

[107] Crane E, Bian Q, Rachel M, Lajoie BR, Wheeler BS, Ralston EJ (2015). Condensin-driven remodelling of X chromosome topology during dosage compensation. Nature.

[108] Shin H, Shi Y, Dai C, Tjong H, Gong K, Alber F (2016). TopDom: an efficient and deterministic method for identifying topological domains in genomes. Nucleic Acids Res.

[109] Oluwadare O, Cheng J (2017). ClusterTAD: an unsupervised machine learning approach to detecting topologically associated domains of chromosomes from Hi-C data. BMC Bioinformatics.

[110] Haddad N, Vaillant C, Jost D (2017). IC-Finder: inferring robustly the hierarchical organization of chromatin folding. Nucleic Acids Res.

[111] Serra F, Baù D, Goodstadt M, Castillo D, Filion GJ, Marti-Renom MA (2017). Automatic analysis and 3D-modelling of Hi-C data using TADbit reveals structural features of the fly chromatin colors. PLoS Comput Biol.

[112] Weinreb C, Raphael BJ (2016). Identification of hierarchical chromatin domains. Bioinformatics.

[113] Lévy-Leduc C, Delattre M, Mary-Huard T, Robin S (2014). Two-dimensional segmentation for analyzing Hi-C data. Bioinformatics.

[114] Yan K-K, Lou S, Gerstein M (2017). MrTADFinder: a network modularity based approach to identify topologically associating domains in multiple resolutions. PLoS Comput Biol.

[115] Norton HK, Emerson DJ, Huang H, Kim J, Titus KR, Gu S (2018). Detecting hierarchical genome folding with network modularity. Nat Methods.

[116] Forcato M, Nicoletti C, Pal K, Livi C, Ferrari F, Bicciato S (2017). Comparison of computational methods for Hi-C data analysis. Nat Methods.

[117] Yu W, He B, Tan K (2017). Identifying topologically associating domains and subdomains by Gaussian mixture model and proportion test. Nat Commun.

[118] Dixon JR, Jung I, Selvaraj S, Shen Y, Antosiewicz-Bourget JE, Lee AY (2015). Chromatin architecture reorganization during stem cell differentiation. Nature.

[119] Dong Q, Li N, Li X, Yuan Z, Xie D, Wang X (2018). Genome-wide Hi-C analysis reveals extensive hierarchical chromatin interactions in rice. Plant J.

[120] Zhao Y-T, Kwon DY, Johnson BS, Fasolino M, Lamonica JM, Kim YJ (2018). Long genes linked to autism spectrum disorders harbor broad enhancer-like chromatin domains. Genome Res.

[121] Duan Z, Andronescu M, Schutz K, McIlwain S, Kim YJ, Lee C (2010). A three-dimensional model of the yeast genome. Nature.

[122] Ay F, Bailey TL, Noble W (2014). Statistical confidence estimation for Hi-C data reveals regulatory chromatin contacts. Genome Res.

[123] Kaul A, Bhattacharyya S, Ay F (2020). Identifying statistically significant chromatin contacts from Hi-C data with FitHiC2. Nat Protoc.

[124] Heinz S, Benner C, Spann N, Bertolino E, Lin YC, Laslo P (2010). Simple combinations of lineage-determining transcription factors prime cis-regulatory elements required for macrophage and B cell identities. Mol Cell.

[125] Mifsud B, Martincorena I, Darbo E, Sugar R, Schoenfelder S, Fraser P (2017). GOTHiC, a probabilistic model to resolve complex biases and to identify real interactions in Hi-C data. PLoS ONE.

[126] Bhattacharyya S, Chandra V, Vijayanand P, Ay F (2019). Identification of significant chromatin contacts from HiChIP data by FitHiChIP. Nat Commun.

[127] Hwang Y-C, Lin C-F, Valladares O, Malamon J, Kuksa PP, Zheng Q (2015). HIPPIE: a high-throughput identification pipeline for promoter interacting enhancer elements. Bioinformatics.

[128] Carty M, Zamparo L, Sahin M, González A, Pelossof R, Elemento O (2017). An integrated model for detecting significant chromatin interactions from high-resolution Hi-C data. Nat Commun.

[129] Xu Z, Zhang G, Jin F, Chen M, Furey TS, Sullivan PF (2016). A hidden Markov random field-based Bayesian method for the detection of long-range chromosomal interactions in Hi-C data. Bioinformatics.

[130] Xu Z, Zhang G, Wu C, Li Y, Hu M (2016). FastHiC: a fast and accurate algorithm to detect long-range chromosomal interactions from Hi-C data. Bioinformatics.

[131] Alinejad-Rokny H, Ghavami R, Rabiee HR, Rezaei N (2020). MaxHiC: robust estimation of chromatin interaction frequency in Hi-C and capture Hi-C experiments. bioRxiv.

[132] Cairns J, Freire-Pritchett P, Wingett SW, Várnai C, Dimond A, Plagnol V (2016). CHiCAGO: robust detection of DNA looping interactions in Capture Hi-C data. Genome Biol.

[133] Ben Zouari Y, Molitor AM, Sikorska N, Pancaldi V, Sexton T (2019). ChiCMaxima: a robust and simple pipeline for detection and visualization of chromatin looping in Capture Hi-C. Genome Biol.

[134] Cao Y, Chen Z, Chen X, Ai D, Chen G, McDermott J (2020). Accurate loop calling for 3D genomic data with cLoops. Bioinformatics.

[135] Vian L, Pękowska A, Rao SSP, Kieffer-Kwon K-R, Jung S, Baranello L (2018). The energetics and physiological impact of cohesin extrusion. Cell.

[136] Robinson MD, McCarthy DJ, Smyth GK (2010). edgeR: a Bioconductor package for differential expression analysis of digital gene expression data. Bioinformatics.

[137] Love MI, Huber W, Anders S (2014). Moderated estimation of fold change and dispersion for RNA-seq data with DESeq2. Genome Biol.

[138] Kingma DP, Ba J. Adam: A method for stochastic optimization. arXiv [cs.LG]. 2014. http://arxiv.org/abs/1412.6980

[139] Genovese CR, Roeder K, Wasserman L (2006). False discovery control with p-value weighting. Biometrika.

[140] Lareau CA, Aryee MJ (2018). hichipper: a preprocessing pipeline for calling DNA loops from HiChIP data. Nat Methods.

[141] Hong H, Jiang S, Li H, Du G, Sun Y, Tao H (2020). DeepHiC: a generative adversarial network for enhancing Hi-C data resolution. PLoS Comput Biol.

[142] Zhang Y, An L, Xu J, Zhang B, Zheng WJ, Hu M (2018). Enhancing Hi-C data resolution with deep convolutional neural network HiCPlus. Nat Commun.

[143] Liu T, Wang Z (2019). HiCNN: a very deep convolutional neural network to better enhance the resolution of Hi-C data. Bioinformatics.

[144] Ester Martin,  Kriegel Hans-Peter, Sander Jiirg, Xu Xiaowei (1996). A density-based algorithm for discovering clusters in large spatial databases with noise.. Kdd.

[145] Kraft K, Magg A, Heinrich V, Riemenschneider C, Schöpflin R, Markowski J (2019). Serial genomic inversions induce tissue-specific architectural stripes, gene misexpression and congenital malformations. Nat Cell Biol.

[146] Royer-Pokora B, Kunkel LM, Monaco AP, Goff SC, Newburger PE, Baehner RL (1986). Cloning the gene for an inherited human disorder—chronic granulomatous disease—on the basis of its chromosomal location. Nature.

[147] Kerem B, Rommens JM, Buchanan JA, Markiewicz D, Cox TK, Chakravarti A (1989). Identification of the cystic fibrosis gene: genetic analysis. Science.

[148] Strathdee CA, Gavish H, Shannon WR, Buchwald M (1992). Cloning of cDNAs for Fanconi’s anaemia by functional complementation. Nature.

[149] Wolffe A (1998). Chromatin: structure and function.

[150] Woodcock CL, Dimitrov S (2001). Higher-order structure of chromatin and chromosomes. Curr Opin Genet Dev.

[151] Li G, Ruan X, Auerbach RK, Sandhu KS, Zheng M, Wang P (2012). Extensive promoter-centered chromatin interactions provide a topological basis for transcription regulation. Cell.

[152] Beagrie RA, Scialdone A, Schueler M, Kraemer DCA, Chotalia M, Xie SQ (2017). Complex multi-enhancer contacts captured by genome architecture mapping. Nature.

[153] Rubin AJ, Barajas BC, Furlan-Magaril M, Lopez-Pajares V, Mumbach MR, Howard I (2017). Lineage-specific dynamic and pre-established enhancer-promoter contacts cooperate in terminal differentiation. Nat Genet.

[154] Montefiori LE, Sobreira DR, Sakabe NJ, Aneas I, Joslin AC, Hansen GT (2018). A promoter interaction map for cardiovascular disease genetics. Elife.

[155] Chen H, Xiao J, Shao T, Wang L, Bai J, Lin X (2019). Landscape of enhancer-enhancer cooperative regulation during human cardiac commitment. Mol Ther Nucleic Acids.

[156] Jung I, Schmitt A, Diao Y, Lee AJ, Liu T, Yang D (2019). A compendium of promoter-centered long-range chromatin interactions in the human genome. Nat Genet.

[157] Lu L, Liu X, Huang W-K, Giusti-Rodríguez P, Cui J, Zhang S (2020). Robust Hi-C maps of enhancer-promoter interactions reveal the function of non-coding genome in neural development and diseases. Mol Cell.

[158] Qin Y, Grimm SA, Roberts JD, Chrysovergis K, Wade PA (2020). Alterations in promoter interaction landscape and transcriptional network underlying metabolic adaptation to diet. Nat Commun.

[159] Lizio M, Harshbarger J, Shimoji H, Severin J, Kasukawa T, Sahin S (2015). Gateways to the FANTOM5 promoter level mammalian expression atlas. Genome Biol.

[160] Bernstein BE, Stamatoyannopoulos JA, Costello JF, Ren B, Milosavljevic A, Meissner A (2010). The NIH roadmap epigenomics mapping consortium. Nat Biotechnol.

[161] Adams D, Altucci L, Antonarakis SE, Ballesteros J, Beck S, Bird A (2012). BLUEPRINT to decode the epigenetic signature written in blood. Nat Biotechnol.

[162] ENCODE Project Consortium (2012). An integrated encyclopedia of DNA elements in the human genome. Nature.

[163] Yue F, Cheng Y, Breschi A, Vierstra J, Wu W, Ryba T (2014). A comparative encyclopedia of DNA elements in the mouse genome. Nature.

[164] Bai X, Shi S, Ai B, Jiang Y, Liu Y, Han X (2020). ENdb: a manually curated database of experimentally supported enhancers for human and mouse. Nucleic Acids Res.

[165] Roadmap Epigenomics Consortium, Kundaje A, Meuleman W, Ernst J, Bilenky M, Yen A (2015). Integrative analysis of 111 reference human epigenomes.. Nature.

[166] Ernst J, Kellis M (2017). Chromatin-state discovery and genome annotation with ChromHMM. Nat Protoc.

[167] Lin CY, Erkek S, Tong Y, Yin L, Federation AJ, Zapatka M (2016). Active medulloblastoma enhancers reveal subgroup-specific cellular origins. Nature.

[168] Ron G, Globerson Y, Moran D, Kaplan T (2017). Promoter-enhancer interactions identified from Hi-C data using probabilistic models and hierarchical topological domains. Nat Commun.

[169] Corradin O, Saiakhova A, Akhtar-Zaidi B, Myeroff L, Willis J, Cowper-Sallari R (2014). Combinatorial effects of multiple enhancer variants in linkage disequilibrium dictate levels of gene expression to confer susceptibility to common traits. Genome Res.

[170] Yao L, Shen H, Laird PW, Farnham PJ, Berman BP (2015). Inferring regulatory element landscapes and transcription factor networks from cancer methylomes. Genome Biol.

[171] Pliner HA, Packer JS, McFaline-Figueroa JL, Cusanovich DA, Daza RM, Aghamirzaie D (2018). Cicero predicts cis-regulatory DNA interactions from single-cell chromatin accessibility data. Mol Cell.

[172] Mehdi T, Bailey SD, Guilhamon P, Lupien M (2019). C3D: a tool to predict 3D genomic interactions between cis-regulatory elements. Bioinformatics.

[173] Zeng W, Wu M, Jiang R (2018). Prediction of enhancer-promoter interactions via natural language processing. BMC Genomics.

[174] Tao H, Li H, Xu K, Hong H, Jiang S, Du G (2021). Computational methods for the prediction of chromatin interaction and organization using sequence and epigenomic profiles. Brief Bioinform.

[175] Pott S, Lieb JD (2014). What are super-enhancers?. Nat Genet.

[176] Oudelaar AM, Davies JOJ, Hanssen LLP, Telenius JM, Schwessinger R, Liu Y (2018). Single-allele chromatin interactions identify regulatory hubs in dynamic compartmentalized domains. Nat Genet.

[177] Quinodoz SA, Ollikainen N, Tabak B, Palla A, Schmidt JM, Detmar E (2018). Higher-order inter-chromosomal hubs shape 3D genome organization in the nucleus. Cell.

[178] Sabari BR, Dall’Agnese A, Boija A, Klein IA, Coffey EL, Shrinivas K (2018). Coactivator condensation at super-enhancers links phase separation and gene control. Science.

[179] Hnisz D, Shrinivas K, Young RA, Chakraborty AK, Sharp PA (2017). A phase separation model for transcriptional control. Cell.

[180] Smith NC, Matthews JM (2016). Mechanisms of DNA-binding specificity and functional gene regulation by transcription factors. Curr Opin Struct Biol.

[181] Wang X, Cairns MJ, Yan J (2019). Super-enhancers in transcriptional regulation and genome organization. Nucleic Acids Res.

[182] Hu Z, Tee W-W (2017). Enhancers and chromatin structures: regulatory hubs in gene expression and diseases. Biosci Rep.

[183] Lee B-K, Jang YJ, Kim M, LeBlanc L, Rhee C, Lee J (2019). Super-enhancer-guided mapping of regulatory networks controlling mouse trophoblast stem cells. Nat Commun.

[184] Nora EP, Lajoie BR, Schulz EG, Giorgetti L, Okamoto I, Servant N (2012). Spatial partitioning of the regulatory landscape of the X-inactivation centre. Nature.

[185] Lovén J, Hoke HA, Lin CY, Lau A, Orlando DA, Vakoc CR (2013). Selective inhibition of tumor oncogenes by disruption of super-enhancers. Cell.

[186] Whyte WA, Orlando DA, Hnisz D, Abraham BJ, Lin CY, Kagey MH (2013). Master transcription factors and mediator establish super-enhancers at key cell identity genes. Cell.

[187] Ing-Simmons E, Seitan VC, Faure AJ, Flicek P, Carroll T, Dekker J (2015). Spatial enhancer clustering and regulation of enhancer-proximal genes by cohesin. Genome Res.

[188] Gong Y, Lazaris C, Sakellaropoulos T, Lozano A, Kambadur P, Ntziachristos P (2018). Stratification of TAD boundaries reveals preferential insulation of super-enhancers by strong boundaries. Nat Commun.

[189] Zhu D-L, Chen X-F, Hu W-X, Dong S-S, Lu B-J, Rong Y (2018). Multiple functional variants at 13q14 risk locus for osteoporosis regulate RANKL expression through long-range super-enhancer: long-range modulation of rankl expression by bmd variants at 13q14.11. J Bone Miner Res.

[190] Hu H, Miao Y-R, Jia L-H, Yu Q-Y, Zhang Q, Guo A-Y (2019). AnimalTFDB 3.0: a comprehensive resource for annotation and prediction of animal transcription factors. Nucleic Acids Res.

[191] Jin J, Tian F, Yang D-C, Meng Y-Q, Kong L, Luo J (2017). PlantTFDB 4.0: toward a central hub for transcription factors and regulatory interactions in plants. Nucleic Acids Res.

[192] Yevshin I, Sharipov R, Kolmykov S, Kondrakhin Y, Kolpakov F (2019). GTRD: a database on gene transcription regulation—2019 update. Nucleic Acids Res.

[193] Jiang Y, Qian F, Bai X, Liu Y, Wang Q, Ai B (2019). SEdb: a comprehensive human super-enhancer database. Nucleic Acids Res.

[194] Khan A, Zhang X (2016). dbSUPER: a database of super-enhancers in mouse and human genome. Nucleic Acids Res.

[195] Wei Y, Zhang S, Shang S, Zhang B, Li S, Wang X (2016). SEA: a super-enhancer archive. Nucleic Acids Res.

[196] Chen C, Zhou D, Gu Y, Wang C, Zhang M, Lin X (2020). SEA version 3.0: a comprehensive extension and update of the Super-Enhancer archive. Nucleic Acids Res.

[197] Hindorff LA, Sethupathy P, Junkins HA, Ramos EM, Mehta JP, Collins FS (2009). Potential etiologic and functional implications of genome-wide association loci for human diseases and traits. Proc Natl Acad Sci U S A.

[198] Freedman ML, Monteiro ANA, Gayther SA, Coetzee GA, Risch A, Plass C (2011). Principles for the post-GWAS functional characterization of cancer risk loci. Nat Genet.

[199] Giral H, Landmesser U, Kratzer A (2018). Into the wild: GWAS exploration of non-coding RNAs. Front Cardiovasc Med.

[200] Dryden NH, Broome LR, Dudbridge F, Johnson N, Orr N, Schoenfelder S (2014). Unbiased analysis of potential targets of breast cancer susceptibility loci by Capture Hi-C. Genome Res.

[201] Martin P, McGovern A, Massey J, Schoenfelder S, Duffus K, Yarwood A (2016). Identifying causal genes at the multiple sclerosis associated region 6q23 using capture Hi-C. PLoS ONE.

[202] McGovern A, Schoenfelder S, Martin P, Massey J, Duffus K, Plant D (2016). Capture Hi-C identifies a novel causal gene, IL20RA, in the pan-autoimmune genetic susceptibility region 6q23. Genome Biol.

[203] Baxter JS, Leavy OC, Dryden NH, Maguire S, Johnson N, Fedele V (2018). Capture Hi-C identifies putative target genes at 33 breast cancer risk loci. Nat Commun.

[204] Welter D, MacArthur J, Morales J, Burdett T, Hall P, Junkins H (2014). The NHGRI GWAS Catalog, a curated resource of SNP-trait associations. Nucleic Acids Res.

[205] Carvalho-Silva D, Pierleoni A, Pignatelli M, Ong C, Fumis L, Karamanis N (2019). Open Targets Platform: new developments and updates two years on. Nucleic Acids Res.

[206] Beck T, Shorter T, Brookes AJ (2020). GWAS Central: a comprehensive resource for the discovery and comparison of genotype and phenotype data from genome-wide association studies. Nucleic Acids Res.

[207] Tian D, Wang P, Tang B, Teng X, Li C, Liu X (2020). GWAS Atlas: a curated resource of genome-wide variant-trait associations in plants and animals. Nucleic Acids Res.

[208] Li MJ, Liu Z, Wang P, Wong MP, Nelson MR, Kocher JPA (2016). GWASdb v2: an update database for human genetic variants identified by genome-wide association studies. Nucleic Acids Res.

[209] Doerge RW (2002). Mapping and analysis of quantitative trait loci in experimental populations. Nat Rev Genet.

[210] GTEx Consortium,  Laboratory, Data Analysis &amp;Coordinating Center (LDACC)—Analysis Working Group,  Statistical Methods groups—Analysis Working Group,  Enhancing GTEx (eGTEx) groups, NIH Common Fund, NIH/NCI (2017). Genetic effects on gene expression across human tissues.. Nature.

[211] McVicker G, van de Geijn B, Degner JF, Cain CE, Banovich NE, Raj A (2013). Identification of genetic variants that affect histone modifications in human cells. Science.

[212] Grubert F, Zaugg JB, Kasowski M, Ursu O, Spacek DV, Martin AR (2015). Genetic control of chromatin states in humans involves local and distal chromosomal interactions. Cell.

[213] Smith AK, Kilaru V, Kocak M, Almli LM, Mercer KB, Ressler KJ (2014). Methylation quantitative trait loci (meQTLs) are consistently detected across ancestry, developmental stage, and tissue type. BMC Genomics.

[214] Nicodemus-Johnson J, Myers RA, Sakabe NJ, Sobreira DR, Hogarth DK, Naureckas ET (2016). DNA methylation in lung cells is associated with asthma endotypes and genetic risk. JCI Insight.

[215] Gate RE, Cheng CS, Aiden AP, Siba A, Tabaka M, Lituiev D (2018). Genetic determinants of co-accessible chromatin regions in activated T cells across humans. Nat Genet.

[216] Greenwald WW, Chiou J, Yan J, Qiu Y, Dai N, Wang A (2019). Pancreatic islet chromatin accessibility and conformation reveals distal enhancer networks of type 2 diabetes risk.. Nature Communications.

[217] Yu J, Hu M, Li C (2019). Joint analyses of multi-tissue Hi-C and eQTL data demonstrate close spatial proximity between eQTLs and their target genes. BMC Genet.

[218] Xia K, Shabalin AA, Huang S, Madar V, Zhou Y-H, Wang W (2012). seeQTL: a searchable database for human eQTLs. Bioinformatics.

[219] Ward LD, Kellis M (2016). HaploReg v4: systematic mining of putative causal variants, cell types, regulators and target genes for human complex traits and disease. Nucleic Acids Res.

[220] Westra H-J, Peters MJ, Esko T, Yaghootkar H, Schurmann C, Kettunen J (2013). Systematic identification of trans eQTLs as putative drivers of known disease associations. Nat Genet.

[221] Gong J, Wan H, Mei S, Ruan H, Zhang Z, Liu C (2019). Pancan-meQTL: a database to systematically evaluate the effects of genetic variants on methylation in human cancer. Nucleic Acids Res.

[222] Zheng Z, Huang D, Wang J, Zhao K, Zhou Y, Guo Z (2020). QTLbase: an integrative resource for quantitative trait loci across multiple human molecular phenotypes. Nucleic Acids Res.

[223] Shumway M, Cochrane G, Sugawara H (2010). Archiving next generation sequencing data. Nucleic Acids Res.

[224] Leinonen R, Akhtar R, Birney E, Bower L, Cerdeno-Tárraga A, Cheng Y (2011). The European nucleotide archive. Nucleic Acids Res.

[225] Nozaki T, Imai R, Tanbo M, Nagashima R, Tamura S, Tani T (2017). Dynamic organization of chromatin domains revealed by super-resolution live-cell imaging. Mol Cell.

[226] Hsu PD, Lander ES, Zhang F (2014). Development and applications of CRISPR-Cas9 for genome engineering. Cell.

[227] Doudna JA, Charpentier E (2014). Genome editing The new frontier of genome engineering with CRISPR-Cas9. Science.

